# The Emerging Role of Large Language Models in Improving Prostate Cancer Literacy

**DOI:** 10.3390/bioengineering11070654

**Published:** 2024-06-27

**Authors:** Marius Geantă, Daniel Bădescu, Narcis Chirca, Ovidiu Cătălin Nechita, Cosmin George Radu, Ștefan Rascu, Daniel Rădăvoi, Cristian Sima, Cristian Toma, Viorel Jinga

**Affiliations:** 1 Department of Urology, “Carol Davila” University of Medicine and Pharmacy, 8 Eroii Sanitari Blvd., 050474 Bucharest, Romaniavioreljinga@yahoo.com (V.J.); 2 Center for Innovation in Medicine, 42J Theodor Pallady Blvd., 032266 Bucharest, Romania; 3 Department of Urology, “Prof. Dr. Th. Burghele” Clinical Hospital, 20 Panduri Str., 050659 Bucharest, Romania; 4 Academy of Romanian Scientists, 3 Ilfov, 050085 Bucharest, Romania

**Keywords:** prostate cancer, ChatGPT, CoPilot, Gemini, cancer literacy, large language models

## Abstract

This study assesses the effectiveness of chatbots powered by Large Language Models (LLMs)—ChatGPT 3.5, CoPilot, and Gemini—in delivering prostate cancer information, compared to the official Patient’s Guide. Using 25 expert-validated questions, we conducted a comparative analysis to evaluate accuracy, timeliness, completeness, and understandability through a Likert scale. Statistical analyses were used to quantify the performance of each model. Results indicate that ChatGPT 3.5 consistently outperformed the other models, establishing itself as a robust and reliable source of information. CoPilot also performed effectively, albeit slightly less so than ChatGPT 3.5. Despite the strengths of the Patient’s Guide, the advanced capabilities of LLMs like ChatGPT significantly enhance educational tools in healthcare. The findings underscore the need for ongoing innovation and improvement in AI applications within health sectors, especially considering the ethical implications underscored by the forthcoming EU AI Act. Future research should focus on investigating potential biases in AI-generated responses and their impact on patient outcomes.

## 1. Introduction

The public launch in November 2022 of ChatGPT, a large language model (LLM) chatbot that can write informed and precise texts on various subjects, including health, has garnered the attention of the medical and health research community. Translation services, chatbots for customer service, and content generation were the first applications that primarily incorporated LLMs. However, their potential in the medical field quickly became apparent. By processing medical literature, patient records, and other forms of data, LLMs have assisted in tasks ranging from drafting medical documents to providing tentative diagnostic suggestions and generating patient-specific medical advice.

Integrating LLMs into healthcare is part of a broader trend toward digitalization and personalized medicine. These models not only enhance the efficiency of healthcare providers but also play a crucial role in democratizing medical knowledge, thus potentially transforming patient outcomes worldwide [1]. Their ability to quickly synthesize and relay complex medical information can improve health literacy among the general public, a critical factor in the prevention and management of diseases [2]. Patients and the general public have begun to use LLMs to seek information about various diseases, which can impact prevention and therapeutic conduct, adherence to treatment, and, ultimately, therapeutic outcomes [3]. The level of health literacy correlates with better therapeutic outcomes [4,5].

Considering the large-scale availability of LLMs and their potential role in the field of health literacy [6,7], this study aimed to evaluate critically, from the perspective of cancer literacy, the performance of three large language models—ChatGPT, Gemini, and CoPilot—compared to the Patient’s Guide on prostate cancer [8,9,10]. Four parameters were considered—accuracy, timeliness, comprehensiveness, and ease of use—for evaluating the answers provided by the three LLMs and the Patient’s Guide to 25 key questions about prostate cancer. For brevity, the research problem can be formulated in the following terms: How effective are three widely available Large Language Models (LLMs)—ChatGPT 3.5, CoPilot, and Gemini—compared to the official Patient’s Guide in delivering accurate, timely, complete, and easy-to-understand information about prostate cancer?

This study makes several key contributions to the field by being the first to conduct a comparative analysis of three widely available Large Language Models (ChatGPT 3.5, CoPilot, and Gemini) against the standard Patient’s Guide in delivering prostate cancer information within Romania’s specific cultural and linguistic context. It uniquely assesses the effectiveness of these LLMs in a non-English speaking environment, filling a significant gap in the literature which primarily focuses on English-centric evaluations. The findings offer actionable insights for healthcare providers and policymakers on using AI to enhance patient education tools, with potential improvements in patient understanding and management of prostate cancer. Furthermore, the study introduces a methodological framework that can be adapted for broader applications in healthcare communication research, setting a new direction for future investigations at the intersection of AI, language technology, and healthcare. In this context, our work aims to cover the research gap in the role of LLMs in improving prostate cancer literacy within a well-defined cultural environment (Romania), by comparing it to the educational tool considered to be the standard, the Patient’s Guide. Although other studies on the role of LLMs for prostate cancer literacy have been published, no research has yet compared the official Patient’s Guide with three freely available LLMs in a linguistically and culturally well-characterized environment.

## 2. Materials and Methods

### 2.1. Study Design and Question Formulation

The methodology for this study was designed to systematically assess the efficacy of three large language models (LLMs: CoPilot, ChatGPT, Gemini) compared to the official Patient’s Guide in providing *accurate*, *timely*, *comprehensive*, and *easy-to-use* information on prostate cancer. We specifically chose to analyze ChatGPT 3.5, CoPilot, and Gemini, due to their distinctive attributes and relevance in the field of AI-driven healthcare communication. ChatGPT 3.5 is distinguished by its advanced natural language processing capabilities, which makes it highly suitable for generating accurate and contextually appropriate responses in the healthcare domain. CoPilot is included for its proficiency in technical support, particularly in interpreting complex medical information and conveying it in a user-friendly manner. Lastly, Gemini is selected for its sophisticated reasoning and decision-making functionalities, which are essential for handling the complex nuances of medical topics such as prostate cancer. These models collectively represent a broad spectrum of the current AI technologies and their varied applications, offering a comprehensive insight into how different AI strategies can enhance patient education. This selection allows us to evaluate the potential of LLMs to improve health literacy, providing valuable data on their strengths and limitations in real-world healthcare settings.

We formulated 25 questions reflecting common queries related to prostate cancer. An English version of the questionnaire (the 25 questions) as well as the raw data frame are freely available [11]. Specifically, in accord with our research objectives, we firstly developed a question bank that covered a broad range of topics related to prostate cancer (i.e., symptoms, diagnostic procedures and screening tests, treatment options, post-treatment care, and lifestyle recommendations). The question bank was validated and refined with the help of clinical experts in oncology and urology. Based on the feedback received from experts, we finalized the set of 25 representative questions. We made sure the questions were diverse enough to thoroughly test the capabilities of the LLMs and the Guide across the four criteria.

On 11 February 2024, the following prompt was used to interrogate the three LLMs: “I am a man, and my doctor has informed me that I have been diagnosed with prostate cancer. I am interested in learning more about the diagnosis, treatment, and overall management of the disease, which will help me better manage the condition and improve my quality of life. Therefore, I have the following questions for which I would like to obtain answers”.

For each question, responses were generated using two general sources: the established official Patient’s Guide and three advanced LLMs—ChatGPT 3.5, Gemini (Pro), and CoPilot (the free version). A single operator queried all three models to ensure consistency in the data collection process. The queries were conducted using incognito mode in Google Chrome to eliminate any personalized search biases, ensuring that each LLM responded based solely on their built-in knowledge and algorithms.

#### 2.1.1. Blinding and Randomization of Responses

After collecting the responses, we performed a randomization process to mix the answers thoroughly. This procedure was to ensure that the subsequent evaluation by experts would be free from preconceived notions about each response’s source. Specifically, we invited the experts to assess the responses without making available the source of the responses. In their grading, the experts did not know whether the responses to the questions were taken from the official Patient’s Guide or generated by LLMs.

#### 2.1.2. Experts and Expert Evaluation

The randomized responses were then presented to a panel of eight experts in prostate cancer (i.e., medical doctors). These experts were affiliated with the foremost hospital in Bucharest, Romania, which is noted for treating the largest number of prostate cancer patients annually. We targeted this hospital to ensure that we had access to the most prominent Romanian medical doctors in this medical field. We sent invitations to all the medical doctors that treat prostate cancer patients and are affiliated with this hospital of interest. Eventually, we ended up with a convenience sample of eight experts.

All experts were males, had an average age of 38.25 years (*SD*: 7.13, *Range*: 20) and an average number of patients per month of 16.88 (*SD*: 25.84, *Range*: 79). We note that the experts displayed a low-to-moderate variability in terms of age (*Coefficient of variation:* 18.63%). On the other hand, they exhibited a high variability in terms of cancer patients treated per month (*Coefficient of variation:* 153.11%); this suggests a highly skewed distribution.

The experts were blinded to the source of each response to maintain the integrity of the assessment process. Furthermore, we implemented this process to reduce the disparities and potential prejudices arising from the variation among medical practitioners in terms of age and the number of cancer patients treated. However, it is important to interpret the data cautiously because the sample is homogenous in terms of sex assigned at birth, with all panel members being male medical practitioners. Due to the limited and newly growing research on this subject, there are no previous studies available regarding the influence of assigned sex at birth on the distribution of answers. However, it is possible that there may be biases in the replies related to this socio-demographic aspect, specifically the sex assigned at birth.

Every member of the panel was provided with a digital version of the questionnaire. Subsequently, we pooled all the responses into a data frame and conducted statistical analysis utilizing the R utilities accessible in RStudio. All participants voluntarily agreed to participate in the study after receiving a consent form. This document provided information on the research’s objectives and context. It also highlighted that participants’ identities would be kept anonymous and their involvement would be treated with utmost confidentiality. Furthermore, it emphasized that participation in the study was entirely voluntary. No incentives, whether monetary or non-monetary, were provided to the research participants. However, we made a commitment to grant them access to the data frame and any scientific documents (such as study reports, scientific publications, oral talks, etc.) that were based on the collected data.

All methods were carried out in accordance with the relevant national and international guidelines and regulations. Informed consent was obtained from all participants. The privacy rights of the study participants were observed.

#### 2.1.3. Evaluation Criteria and Scoring

Each expert independently evaluated the responses based on four key criteria (ATCE algorithm): accuracy, timeliness, comprehensiveness, and ease of use [12,13,14,15]. Each criterion was rated on a Likert scale ranging from 1 (poor) to 5 (excellent). This scoring system allowed us to quantitatively assess the quality and utility of the information provided by each source [16].

#### 2.1.4. Language and Cultural Considerations

The entire evaluation process was conducted in Romanian, which not only facilitated a natural understanding among the native expert panel but also enabled an assessment of how effectively the LLMs could handle and reflect local and cultural nuances in their responses. This approach will inform the future development of ethical, diverse, equitable, and inclusive human-LLM collaborative models to improve literacy concerning prostate cancer.

### 2.2. Statistical Analysis

We implemented a range of statistical techniques that were appropriate for achieving the goals of our study. Specifically, our interest was in determining (a) if the Guide surpassed each of the three LLMs, and (b) which information source was the most effective in the context of our study design.

We aggregated the scores assigned by each of the eight experts to each of the 25 questions by sources (tools) for information. Firstly, we performed the aggregations per criterion (accuracy, timeliness, comprehensiveness, and ease of use). Then, we performed a grand aggregation, i.e., we computed the sum of all scores irrespective of the four criteria). We fit linear mixed-effects models by Restricted Maximum Likelihood (REML) to the resulting aggregation of scores. We selected this family of models as we wanted to control for the variations attributable to differences across experts, i.e., observable differences such as age, number of patients, and latent differences. This family of statistical models is useful as it allows for separating the fixed effects (the differences among the ratings given by the experts) from the random effects (modeling the dependence and independence among data points due to the grouping structure, i.e., measurements for each information source are grouped by expert). We performed the test of the mixed-effects models using the algorithms implemented in the *lme4* R package (version 1.1-35.4). Also, we performed pairwise comparisons using the *emmeans* R package (version 1.10.2). Specifically, we were interested in comparing information sources with the purpose to ascertain the performance of each tool in relation to the others (e.g., ChatGPT vs. CoPilot, ChatGPT vs. the Guide, ChatGPT vs. Gemini, etc.).

Before running the statistical analysis (fitting the mixed-effects models and performing pairwise comparisons), we checked our data for different assumptions. Firstly, we tested the assumption of normality of the residuals using the Shapiro–Wilk test (i.e., whether the score distribution for each source of information deviated from a normal distribution). Secondly, we tested for the assumption of the homogeneity of variances. For this purpose, we used Levene’s test (the *car* R package (Version 3.1-2)) to understand if the scores were equal across different levels of the sources of information. Additionally, we used other two similar tests: Bartlett’s test (this tends to be more robust when data are normally distributed) and the Fligner–Killeen test (this is less sensitive to the normality of distributions). We used Levene’s Test, Bartlett’s test, and the Fligner–Killeen test to reach a more comprehensive overview of variance homogeneity and to provide solid grounds for parametric test application. Each medical specialist gave complete responses to the 25 queries, which resulted in no missing data. For replication purposes, the code and the data are freely available [11].

## 3. Results

In Table 1, we report the aggregated distribution of ratings (grades or scores) that panel members gave to each information source. We provide the distribution of total scores per each assessment criterion (accuracy, timeliness, comprehensiveness, ease of use) and the grand total of scores (accounting for all the criteria). From a technical perspective, each of the dimensions (accuracy is one of them) illustrated in Table 1 can be up to 125 (i.e., there are 25 questions and each question is assessed on a Likert scale from 1 to 5). The range of scores given by the eight experts suggests that the Guide does not approach the theoretical maximum of 125. From a substantial perspective, we may suspect that experts display the corresponding range of scores (62; 102), because the Guide could not include sufficient information due to being limited by the print space (the Guide version in the references is the PDF version of the printed format, the one distributed to patients with prostate cancer). This indicates the potential of LLMs to enhance the traditional, printed offering of information about prostate cancer.

Table 2 illustrates the results of five linear mixed-effects models that fit the data structure presented in Table 1. We fit these models to understand how the panel experts rated the four specific sources (i.e., ChatGPT, CoPilot, Gemini, and the Guide) and their effectiveness in providing information related to prostate cancer. The Guide (or the assessments associated with the information conveyed by the Guide) stands as the baseline in all the models reported in Table 2.

We fit Model 1 on the total scores elicited by the panel experts (this corresponds to the *Grand total* in Table 1). According to this model, there is significant variation among experts, indicating differing baseline opinions. However, the intercept (Est. 361.50, *p* < 0.001) shows a high score that, on its own, indicates the Guide to be a fairly effective information source. In other words, this sets a high standard for the other sources or tools. ChatGPT (Est. 55.00, *p* < 0.001) and CoPilot (Est. 35.75, *p* < 0.01) provide statistically significant improvements over the Guide, indicating their additional benefits. At the same time, Gemini does not significantly alter the perception (Est. = 0.25, *p* = 0.98), suggesting it offers no improvement over the Guide.

The results corresponding to Model 2 reveal a lower variance than Model 1, indicating more consistency in expert opinions for the *accuracy* criterion. ChatGPT (Est. 15.50, *p* < 0.000) and CoPilot (Est. 8.25, *p* < 0.01) are valuable in terms of the accuracy of the information provided. Again, ChatGPT is particularly influential for this specific criterion. Model 3 exhibits variability among experts concerning the *timeliness* of the responses generated by the four sources of information. Furthermore, ChatGPT provides a consistent improvement compared to the baseline (Est. 7125, *p* < 0.05).

Model 4 displays the lowest variability, indicating strong consensus among experts regarding the *comprehensiveness* dimension of the responses. CoPilot (Est. 14,625, *p* < 0.001) and ChatGPT (Est. 20,500, *p* < 0.000) are seen as highly effective, with ChatGPT showing the most substantial positive effect. Model 5 indicates a moderate consensus among experts concerning the *ease-of-use* evaluation dimension. As in the previous models, ChatGPT (Est. 11,875, *p* < 0.01) and CoPilot (Est. 10,000, *p* < 0.05) enhance ratings significantly.

As a general commentary, ChatGPT consistently emerges as the most effective source across different criteria, receiving high ratings from panel experts. This suggests its robustness and reliability as a source of prostate cancer information. CoPilot is favorably viewed, though its impact is slightly less pronounced than that of ChatGPT. However, experts still consider it a valuable tool. Gemini is viewed either neutrally or negatively across models. This suggests that while it may have uses, it might not be the best source for disseminating prostate cancer information.

In all the models, the Guide (as baseline) remains consistently high, suggesting it is a robust tool across various specific criteria. While the Guide is practical, ChatGPT and CoPilot introduce additional features or present information in a way that the experts find even more helpful or accessible. Gemini presents a non-significant effect (except for Model 2, where it is negative) that suggests it does not consistently offer improvements over the Guide.

Table 3 reports a series of pair-wise comparisons between the information tools that the experts evaluated. These post hoc tests are necessary to indicate which tools differ from each other and how. As indicated in Table 3, we associate these post hoc tests with the linear mixed-effects models reported in Table 2.

Based on the information available in Table 3, we state that, across all models, ChatGPT consistently emerges as the most effective tool, often showing significant improvements over the Guide and other tools. CoPilot performs better than the Guide and is comparable to other tools but does not consistently surpass ChatGPT. Gemini shows the least consistent performance, often not significantly better than the Guide, and is usually less effective than CoPilot and ChatGPT.

For instance, the post hoc tests corresponding to Model 1 illustrate that ChatGPT is significantly more effective than the Guide (Est. −55.00, *p* < 0.001), followed by Gemini (Est. −54.75, *p* < 0.001). Even if there is no significant difference between ChatGPT and CoPilot (Est. −19.35, *p* = 0.36), the numerical difference marks a slight preference for ChatGPT among the experts.

Table 4 reports the results of the tests used to assess the normality of data distributions and the homogeneity of variances across the different groups represented by each source of information. We used the Shapiro–Wilk test (W, the assumption of normality), Levene’s test, Bartlett’s K-squared test, and the Fligner–Killeen test (homogeneity of variance). All tools (sources of information) show *p*-values well above 0.05, suggesting that the scores are normally distributed for each tool. The consistent results across Levene’s, Bartlett’s, and the Fligner–Killeen tests indicate that the assumption of equal variances holds true for all categories. These diagnostics support the use of linear mixed-effects models that were reported in Table 2.

## 4. Discussion

This study’s exploration of Large Language Models (LLMs) such as ChatGPT, Gemini, and CoPilot has yielded significant insights into their potential to enhance cancer literacy, particularly within prostate cancer and specific cultural contexts. The findings reveal varying degrees of effectiveness among these models in improving prostate cancer information and literacy among patients.

Among the three LLMs evaluated, ChatGPT and CoPilot performed better than the third LLM, Gemini, and outperformed the traditional Patient’s Guide across all evaluated criteria. Statistically significant differences between ChatGPT and CoPilot were not observed, indicating comparable performance levels between these two models. The results are aligned with previous data on the efficacy of the LLMs ChatGPT and CoPilot (formerly Bard) in providing accurate, timely, complete, and easy-to-understand information about prostate cancer [17].

The results underscore the potential of LLMs to enhance the effectiveness of patient and caregiver education regarding prostate cancer. The study demonstrates that, for prostate cancer, there are statistically significant differences between the LLMs, with ChatGPT and CoPilot emerging as superior sources of LLM-based information. Concurrently, ChatGPT and CoPilot are identified as prime candidates for developing personalized virtual assistants [18] to aid patients diagnosed with prostate cancer and their families.

Traditional patient and family education methods [19] like the Patient’s Guide could also benefit from developing LLMs. In the future, LLMs could contribute to creating dynamic guides that offer higher accuracy and more current and consistent information, and that are more accessible for patients and their families to understand, co-created by physicians and patients [20,21]

It is acknowledged that using LLMs raises ethical questions [22], particularly concerning the accuracy of machine-generated advice and its impact on patient decision-making. The role of physicians [23] is essential in ensuring the reliability of these tools and establishing clear guidelines for their use to prevent misinformation and ensure the quality of information delivered to patients and families. For these reasons, the development of a human–LLM collaborative model is crucial [24]. In the AI era, the traditional linear model of physician–patient communication [25] is transforming into a complex and dynamic model [26] where the professional authority (the physician) must actively and continuously contribute to developing, training, and refining LLM-based chatbots. At the same time, the beneficiary (the patient and family) evolves from a passive recipient of information into an active contributor.

Our study makes a significant research contribution as it is the first to assess prostate cancer literacy in terms of accuracy, timeliness, comprehensiveness, and usability of the official Patient’s Guide alongside three LLMs within a well-defined cultural context (Romanian language, experts from the most relevant hospital specializing in prostate cancer management). The findings underscore the specific roles that ChatGPT and CoPilot could play in enhancing the effectiveness of communicating prostate cancer information to patients in this specific environment.

### 4.1. Limitations

Our study entails several limitations that warrant consideration. Firstly, the assessments of the LLMs and the national guide by medical oncologists, despite their expertise, remain susceptible to subjectivity and individual biases. The diversity and size of the expert panel may also affect the generalizability of our findings, as it may not adequately represent the broader oncological community. Additionally, the dynamic nature of LLM technologies means that our findings could become outdated as these models evolve. The complexity of prostate cancer as a medical condition poses another significant challenge, as it demands comprehensive information that may not be fully captured by the selected evaluation criteria of accuracy, timeliness, comprehensiveness, and ease of use. These factors should be carefully considered when interpreting the study outcomes and planning future research.

### 4.2. Future Directions

There is immense potential for integrating LLMs more deeply into the healthcare system. Developing models that can interact seamlessly with electronic health records (EHRs) to provide contextual advice could revolutionize patient care [27,28,29]. Additionally, further research should focus on personalizing LLMs’ interactions based on individual patient histories to enhance the relevance and effectiveness of the information provided. This underscores the need for regulatory frameworks to oversee the deployment of LLMs in healthcare settings [30]. Such regulations should ensure these tools meet stringent accuracy and safety standards, like other medical devices. The conclusions of the study resonate with the recently approved EU AI Act [31] that will be effective from 2026, a key document highlighting the need for expert oversight of high-risk AI systems such as the LLMs used in the health contexts.

Our findings suggest that the Guide is a solid foundation for providing information about prostate cancer. However, ChatGPT and CoPilot present enhancements that recommend their incorporation in information dissemination strategies, possibly making the information more engaging, accessible, or comprehensive. Decisions about which tool to use or recommend should consider these differences in effectiveness. Tools that significantly improve the Guide could be prioritized for situations requiring higher engagement or more profound understanding. Understanding that Gemini does not improve upon the Guide might lead to reconsidering its use or pushing for its development to meet the guidelines and other tools.

In summary, while the Guide sets a high standard of effectiveness, the additional benefits provided by ChatGPT and CoPilot underline the importance of continuous improvement and innovation in educational tools, especially in critical health information domains like prostate cancer.

Our results can guide healthcare providers, researchers, and decision-makers in optimizing the tools and resources they deploy for education and communication about prostate cancer, ensuring that the most effective platforms are utilized to disseminate crucial health information.

## 5. Conclusions

Although the Guide is currently considered the standard for communicating information about prostate cancer to Romanian patients, its performance is deemed suboptimal according to expert scores, limiting full patient benefit. ChatGPT and Co-Pilot have the potential to enhance the Guide’s effectiveness through a human-supervised model of collaboration.

As these models continue to evolve, their influence on the medical field is expected to grow, making their study and understanding an essential area of research. The use of LLMs like ChatGPT and CoPilot in improving cancer literacy among prostate cancer patients holds promising potential. However, continuous improvements, rigorous testing, and thoughtful integration into clinical practice, accompanied by appropriate ethical and regulatory oversight, are essential to fully realize their benefits without compromising patient safety or quality of care.

## Figures and Tables

**Table 1:** Distributions of aggregated scores given by panel experts.

Criterion	Expert ID	ChatGPT	Gemini	CoPilot	Guide
*Grand total*					
	1	432	373	432	409
	2	408	275	335	249
	3	377	341	377	343
	4	434	394	410	376
	5	411	388	394	372
	6	456	363	416	359
	7	451	406	448	435
	8	363	354	366	349
*Accuracy*					
	1	109	93	105	101
	2	99	66	78	62
	3	96	80	93	81
	4	112	101	101	101
	5	100	96	95	88
	6	113	91	106	90
	7	109	96	106	102
	8	88	79	84	77
*Timeliness*					
	1	108	93	110	104
	2	100	66	77	66
	3	98	80	101	98
	4	112	101	103	104
	5	99	96	93	94
	6	109	91	100	95
	7	112	96	115	114
	8	92	79	97	98
*Comprehensiveness*					
	1	98	78	102	90
	2	101	63	86	58
	3	94	74	90	78
	4	106	87	96	80
	5	97	89	93	82
	6	118	79	106	78
	7	109	89	107	99
	8	93	86	89	87
*Ease of use*					
	1	117	108	115	114
	2	108	75	94	63
	3	89	93	93	86
	4	104	105	110	91
	5	115	109	113	108
	6	116	100	104	96
	7	121	113	120	120
	8	90	95	96	87

Note: We aggregated experts’ ratings (1 to 5) across all 25 questions. The *Grand total* is computed over all the aggregations performed across the four assessment criteria: *accuracy*, *timeliness*, *comprehensiveness*, and *ease of use*.

**Table 2:** Linear mixed-effects models fit by REML.

Model 1: General						
*Random effects*	Variance	Std. Dev.				
Groups (intercept)	1266.8	35.59				
Residual	531.8	23.06				
*Fixed intercepts*						
	Estimate	SE	df	t value	Pr(>|t|)	
(Intercept)	361.50	14.99	11.25	24,109	0.000000	***
CoPilot	35.75	11.53	21.00	3100	0.005418	**
Gemini	0.25	11.53	21.00	0.022	0.982907	
ChatGPT	55.00	11.53	21.00	4770	0.000103	***
Model 2: Accuracy						
*Random effects*	Variance	Std. Dev.				
Groups (intercept)	105.11	10.252				
Residual	26.27	5.125				
*Fixed intercepts*						
	Estimate	SE	df	t value	Pr(>|t|)	
(Intercept)	87.80	4.05	9.59	21,654	21.00	***
CoPilot	8.25	2.56	21.00	3219	0.00411	**
Gemini	0.00	2.56	21.00	0.000	100000	
ChatGPT	15.50	2.56	21.00	6049	0.00000	***
Model 3: Timeliness						
*Random effects*	Variance	Std. Dev.				
Groups (intercept)	88.73	9.419				
Residual	40.95	6.399				
*Fixed intercepts*						
	Estimate	SE	df	t value	Pr(>|t|)	
(Intercept)	96,625	11,645	24,000	21,654	0.0000	***
CoPilot	2875	21,000	0.899	3219	0.3791	
Gemini	−8875	21,000	−2774	0.000	0.0114	*
ChatGPT	7125	21,000	2227	6049	0.0370	*
Model 4: Comprehensiveness						
*Random effects*	Variance	Std. Dev.				
Groups (intercept)	39.37	6.274				
Residual	50.66	7.118				
*Fixed intercepts*						
	Estimate	SE	df	t value	Pr(>|t|)	
(Intercept)	81,500	3355	17,793	24,295	0.0000	***
CoPilot	14,625	3559	21,000	4110	0.0005	***
Gemini	−0.875	3559	21,000	−0.246	0.8082	
ChatGPT	20,500	3559	21,000	5760	0.0000	***
Model 5: Ease of use						
*Random effects*	Variance	Std. Dev.				
Groups (intercept)	132.68	11.518				
Residual	52.87	7.271				
*Fixed intercepts*						
	Estimate	SE	df	t value	Pr(>|t|)	
(Intercept)	95,625	4816	11,050	19,856	0.00000	***
CoPilot	10,000	3636	21,000	2751	0.01198	*
Gemini	4125	3636	21,000	1135	0.26932	
ChatGPT	11,875	3636	21,000	3266	0.00369	**

Note. In each model, we have 32 observations and eight experts. The *t*-tests use Satterthwaite’s method. The Guide is the baseline in each model. * *p* < 0.05, ** *p* < 0.01, *** *p* < 0.001.

**Table 3:** Post hoc tests for comparing sources of information.

Contrast	Estimate	SE	df	t.Ratio	*p* Value
*Model 1: General*					
Guide–CoPilot	−35.75	11.05	21	−3.100	0.0257
Guide–Gemini	−0.25	11.05	21	−0.022	>0.9999
Guide–ChatGPT	−55.00	11.05	21	−4.770	0.0006
CoPilot–Gemini	35.50	11.05	21	3.079	0.0270
CoPilot–ChatGPT	−19.25	11.05	21	−1.669	0.3638
Gemini–ChatGPT	−54.75	11.05	21	−4.748	0.0006
*Model 2: Accuracy*					
Guide–CoPilot	−8.25	2.56	21	−3.219	0.0198
Guide–Gemini	0.00	2.56	21	0.000	>0.9999
Guide–ChatGPT	−15.50	2.56	21	−6.049	<0.0001
CoPilot–Gemini	8.25	2.56	21	3.219	0.0198
CoPilot–ChatGPT	−7.25	2.56	21	−2.829	0.0458
Gemini–ChatGPT	−15.50	2.56	21	−6.049	<0.0001
*Model 3: Timeliness*					
Guide–CoPilot	−2.88	3.2	21	−0.899	0.8057
Guide–Gemini	8.88	3.2	21	2.774	0.0514
Guide–ChatGPT	−7.12	3.2	21	−2.227	0.1485
CoPilot–Gemini	11.75	3.2	21	3.672	0.0072
CoPilot–ChatGPT	−4.25	3.2	21	−1.328	0.5559
Gemini–ChatGPT	−16.00	3.2	21	−5.001	0.0003
*Model 4: Comprehensiveness*					
Guide–CoPilot	−14.625	3.56	21	−4.110	0.0026
Guide–Gemini	0.875	3.56	21	0.246	0.9946
Guide–ChatGPT	−20.500	3.56	21	−5.760	0.0001
CoPilot–Gemini	15.500	3.56	21	4.355	0.0015
CoPilot–ChatGPT	−5.875	3.56	21	−1.651	0.3734
Gemini–ChatGPT	−21.375	3.56	21	−6.006	<0.0001
*Model 5: Ease of use*					
Guide–CoPilot	−10.00	3.64	21	−2.751	0.0539
Guide–Gemini	−4.12	3.64	21	−1.135	0.6729
Guide–ChatGPT	−11.88	3.64	21	−3.266	0.0179
CoPilot–Gemini	5.88	3.64	21	1.616	0.3915
CoPilot–ChatGPT	−1.88	3.64	21	−0.516	0.9544
Gemini–ChatGPT	−7.75	3.64	21	−2.132	0.1757

**Table 4:** Tests for normality and homogeneity of variances.

*Normality Assumption*	*Homogeneitey of Variances*
*Overall evaluation*	
Guide, W = 0.91237, *p* = 0.3711	Levene’s test: F(3, 28) = 0.1927, *p* = 0.9005
CoPilot, W = 0.98284, *p* = 0.9756	Bartlett’s K-squared = 1.945, df = 3, *p* = 0.5839
Gemini, W = 0.88878, *p* = 0.2280	Fligner–Killeen test: med chi-squared = 0.28729, df = 3, *p* = 0.9624
ChatGPT, W = 0.93274, *p* = 0.5414	
*Accuracy evaluation*	
Guide, W = 0.90624, *p* = 0.3284	Levene’s test: F(3, 28) = 0.3158, *p* = 0.8138
CoPilot, W = 0.88498, *p* = 0.2100	Bartlett’s K-squared = 1.4537, df = 3, *p* = 0.693
Gemini, W = 0.90508, *p* = 0.3207	Fligner–Killeen test: med chi-squared = 0.97968, df = 3, *p* = 0.8062
ChatGPT, W = 0.91362, *p* = 0.3802	
*Timeliness evaluation*	
Guide, W = 0.83798, *p* = 0.0718	Levene’s test: F(3, 28) = 0.1206, *p* = 0.9472
CoPilot, W = 0.94442, *p* = 0.6551	Bartlett’s K-squared = 2.4542, df = 3, *p* = 0.4836
Gemini, W = 0.90508, *p* = 0.3207	Fligner–Killeen test: med chi-squared = 0.19041, df = 3, *p* = 0.9791
ChatGPT, W = 0.90091, *p* = 0.2944	
*Comprehensiveness*	
Guide, W = 0.92876, *p* = 0.5048	Levene’s test: F(3, 28) = 0.1023, *p* = 0.9580
CoPilot, W = 0.91887, *p* = 0.4207	Bartlett’s K-squared = 1.2837, df = 3, *p* = 0.733
Gemini, W = 0.87805, *p* = 0.1804	Fligner–Killeen test: med chi-squared = 0.090494, df = 3, *p* = 0.993
ChatGPT, W = 0.91983, *p* = 0.4285	
*Easy-to-use*	
Guide, W = 0.95716, *p* = 0.7827	Levene’s test: F(3, 28) = 0.675, *p* = 0.5746
CoPilot, W = 0.91231, *p* = 0.3706	Bartlett’s K-squared = 2.4896, df = 3, *p* = 0.4772
Gemini, W = 0.90322, *p* = 0.3088	Fligner–Killeen test: med chi-squared = 1.4162, df = 3, *p* = 0.7017
ChatGPT, W = 0.87014, *p* = 0.1512	

Note. We used the Shapiro–Wilk test (W) for checking the normality assumption.

## Data Availability

The data, the questionnaire, the code, and other related files are freely available for replication and secondary data analysis at https://doi.org/10.5281/zenodo.11217682, Accessed on 20 May 2024.
